# A new method to evaluate glenoid erosion in instable shoulder

**DOI:** 10.1186/1755-7682-6-42

**Published:** 2013-10-18

**Authors:** Roberto Y Ikemoto, Joel Murachovsky, Luis G Nascimento, Rogerio S Bueno, Luiz Henrique de Oliveira, Edson N Fujiki, Luiz Carlos de Abreu, Vitor E Valenti, Sergio L Checchia

**Affiliations:** 1 Department of Orthopaedics and Traumatology, Faculdade de Medicina do ABC, Av. Príncipe de Gales, 821., 09060-650 Santo Andre, SP, Brazil; 2 Department of Orthopaedics and Traumatology, Faculdade de Ciências Médicas, Santa Casa de Misericórdia de São Paulo (SCMSP), Av. Príncipe de Gales, 821., 09060-650 Santo Andre, SP, Brazil; 3 Faculty of Philosophy and Sciences, UNESP, Av. Hygino Muzzi Filho, 737., Marilia, SP 17.525-900, Brazil

**Keywords:** Shoulder joint, Arthroscopy, Tomography, Glenoid cavith, Grafting bone

## Abstract

**Background:**

We aimed to establish values and parameters using multislice reconstruction in axial computerized tomography (CT) in order to quantify the erosion of the glenoid cavity in cases of shoulder instability.

**Methods:**

We studied two groups using CT. Group I had normal subjects and Group II had patients with shoulder instability. We measured values of the vertical segment, the superior horizontal, medial and inferior segments, and also calculated the ratio of the horizontal superior and inferior segments of the glenoid cavity in both normal subjects and those with shoulder instability. These variables were recorded during arthroscopy for cases with shoulder instability.

**Results:**

The mean values were 40.87 mm, 17.86 mm, 26.50 mm, 22.86 mm and 0.79 for vertical segment, the superior horizontal, medial and inferior segments, and the ratio between horizontal superior and inferior segments of the glenoid cavity respectively, in normal subjects. For subjects with unstable shoulders the mean values were 37.33 mm, 20.83 mm, 23.07 mm and 0.91 respectively. Arthroscopic measurements yielded an inferior segment value of 24.48 mm with a loss of 2.39 mm (17.57%). The ratio between the superior and inferior segments of the glenoid cavity was 0.79. This value can be used as a normative value for evaluating degree of erosion of the anterior border of the glenoid cavity. However, values found using CT should not be used on a comparative basis with values found during arthroscopy.

**Conclusions:**

Computerized tomographic measurements of the glenoid cavity yielded reliable values consistent with those in the literature.

## Background

Shoulder instability is one of the most challenging problems involving the shoulder joint due to its frequency and variety of anatomic and pathologic alterations, but mainly because of the high risk of recurrence even after surgical repair [1,2]. The failure rate of open surgery (until very recently considered the gold standard for treatment of this disorder) is on average 4%, ranging from 0 to 11% in the literature [3]. Arthroscopy surgery was developed to minimize the surgical morbidity of open surgery and to improve functional treatment outcomes. The development of new materials and instruments allied with new surgical arthroscopic techniques have resulted in better outcomes with fewer recurrences, equivalent to results achieved using “open” surgical methods [1].

Although the risk of recurrence of post surgical instability is low whether open or by arthroscopic surgery, this procedure still leaves the surgeon somewhat apprehensive as to the final results. Treatment of shoulder instability should be based on reconstruction of the stabilizing mechanisms [4]. Restoration of these equilibrating factors and of the movement arc of the shoulder is important to enable the return to normal activities, particularly for young patients who engage in sports [2].

Among the anatomic and pathologic alterations resulting in shoulder instability, erosion of the anterior border of the glenoid cavity is considered one of the factors responsible for recurrence of the problem and should be diagnosed and treated adequately [1]. According to Sugaya et al. [5], due to the lack of a practical and precise method to quantify these lesions, there is no consensus on the magnitude of erosion that would justify the use of a bone implant. Different indexes and values exist in the literature regarding the indication for treatment of erosion of the anterior border of the glenoid cavity, such as percentage area eroded versus joint surface area, width or height of the glenoid cavity [6].

In view of the above considerations, we aimed to establish values and parameters for the erosion of the anterior border of the glenoid cavity in individuals who had traumatic shoulder instability.

## Methods

This is a case–control, diagnostic study, undertaken from July to December 2006 in a public hospital in São Paulo, Brazil, with patients with shoulder instability due to trauma and healthy controls. The study was approved by the local Research Ethics Committee of the Medical Faculty of ABC and patients and controls signed informed consent forms.

### Subjects

Group 1 comprised 50 volunteers (100 shoulders evaluated). Members of this group had no history of shoulder articulation problems and a mean age of 25.58 years (range 18–44 years), and were invited for the study when visiting the hospital for reasons other than shoulder problems. They had no previous history of shoulder diseases. These patients were from lower limbs and back pain complains ambulatory without other clinical pathologies or under pharmacological treatments. Thirty-four individuals of this group were male and 16 were female, 40 were right-handed and 10 left-handed.

Group 2 comprised 23 patients (23 shoulders), who were admitted in the Shoulder Group Clinic with shoulder traumatic instability. This diagnosis was reached based on the history of the first traumatic occurrence reported by the patient and a clinical exam, which revealed no capsular ligamentous laxity. Radiographic exams, consisting of a corrected frontal view, scapular profile, axillary and de Bernargeau views, revealed no fractures or erosion of the glenoid cavity in any of these subjects.

The mean age of this group was 23 years (range 19–51 years) and there were 20 males and two female subjects, 21 right-handed and two left-handed. The right shoulder was affected in 8 cases and the left shoulder in 15 cases. The affected side was the dominant right side in only 6 cases. Dominance was not significantly different between groups. On study entry, the mean number of episodes of instability was 9.9 (range 2–40).

In group 2, the glenoid cavity was also measured during arthroscopy.

### Tomographic examination

We measured the glenoid cavity in the two groups groups using multislice CT with a three-dimensional reconstruction feature of the software series V1.76*R002, Alato - STD, CTO 10ª, Option Key - Realview. A Toshiba, model Asteion TSX – 021B (2K201065E*B) was used for tomography in all exams. One trained radiologist indicated the reference points in the images in all cases.

All subjects (with or without a history of shoulder instability) underwent tomography of both shoulders. The dorsal decubitus position was used, with the superior members positioned beside the body and in neutral arm rotation.

Axial cuts of 0.3 mm were done for three-dimensional reconstruction on coronal and sagital planes for calculation of the volume. Imaging reconstruction was performed at intervals of 1.5 mm.

**Figure 1:**
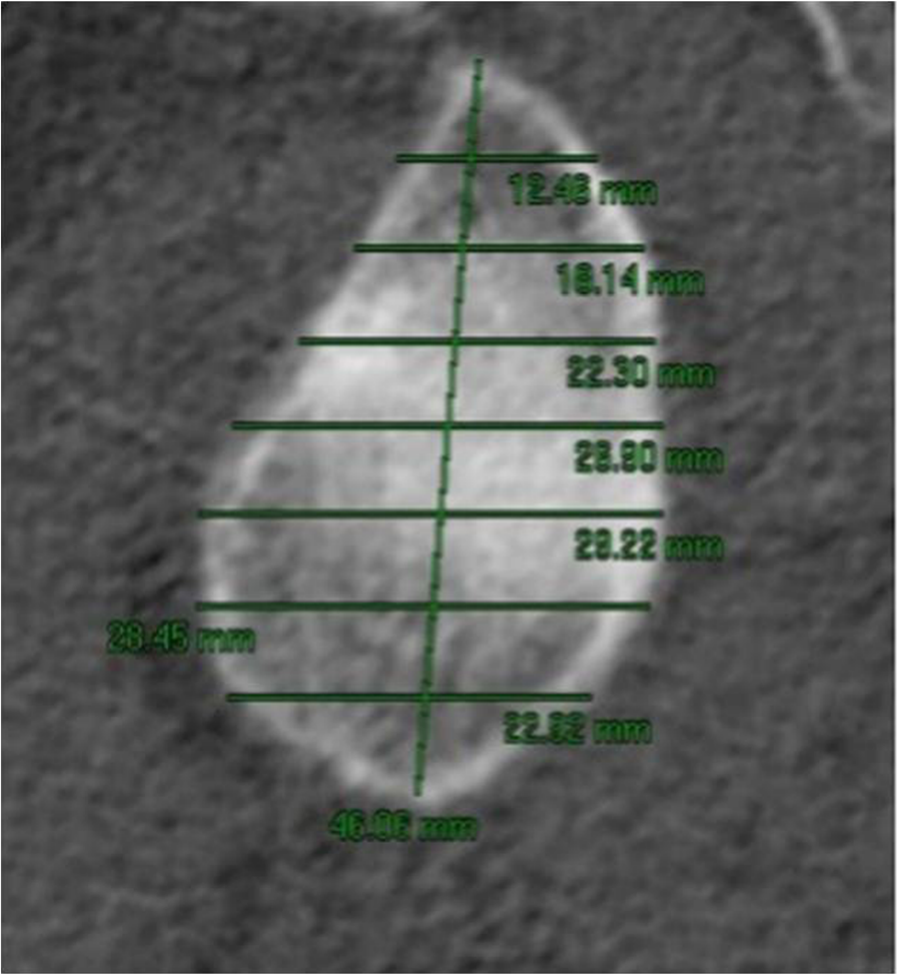
Image of sagital slice of the scapula on the face of glenoid cavity, with segments marked.

Firstly, we traced a straight line from the supraglenoid to the infraglenoid tubercle, which was called the Vertical Segment (VS). This straight line was then divided into seven segments and, from each of the seven points, perpendicular lines were drawn from the anterior to the posterior border of the glenoid cavity (called segments 1,2,3,4,5,6 and 7) and measured (Figure 1). For statistical evaluations only values for the Vertical Segment, and segments 2, 4 and 6 were used, corresponding to the values needed to calculate the ratio and establish values for comparison with the data in the literature.

The ratio of segment 2 to segment 6 was used, based on the assumption that the superior portion of segment 2 would be constant, in spite of glenoid cavity erosion, while segment 6 represented the eroded portion.

### Arthroscopic evaluation

From 21 to 45 days after tomographic measurements, cases with shoulder instability underwent reparative arthroscopic surgery. The decubitus lateral position was used in all cases, with longitudinal traction and the use of two anterior ports (anterior-superior and anterior inferior) as well as a posterior port.

**Figure 2:**
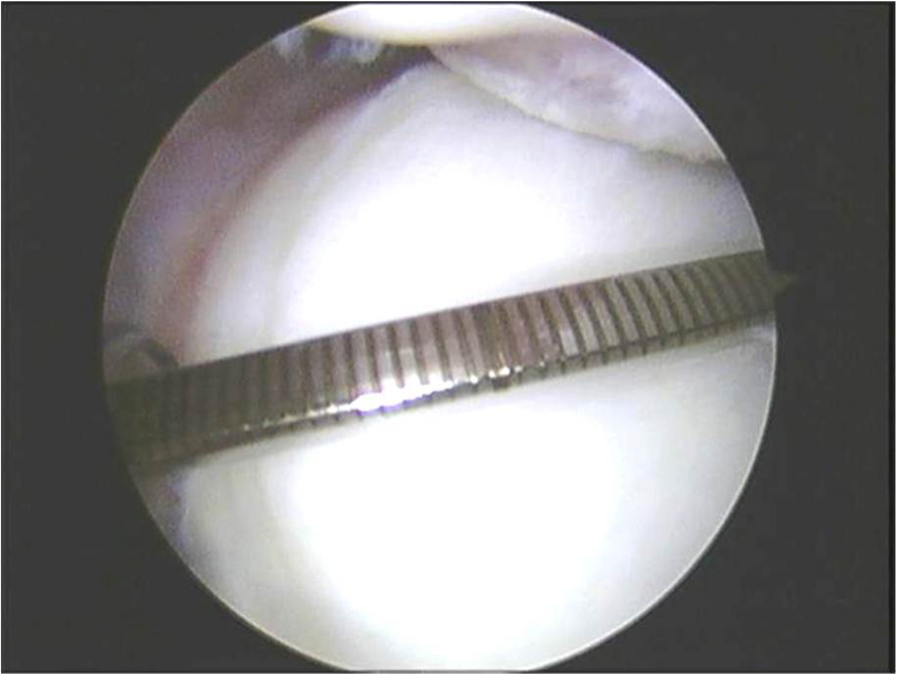
Computed tomography showing the glenoid with the measurements of each segment.

**Figure 3:**
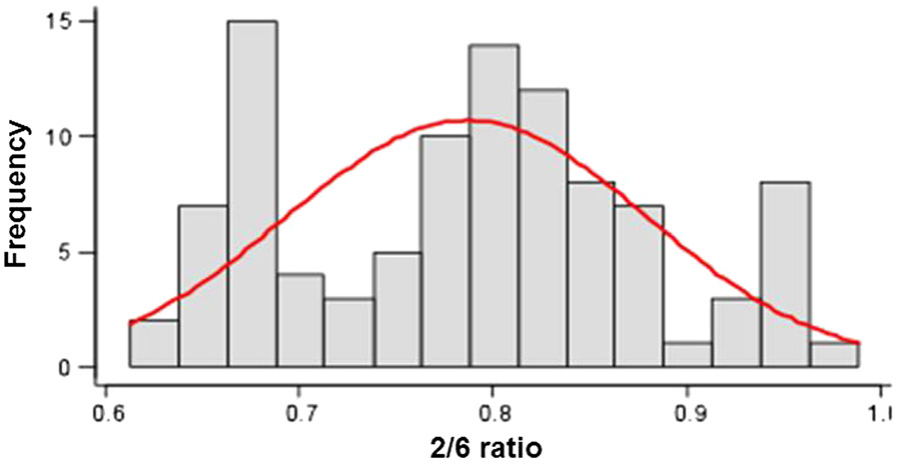
Photograph of measurement of glenoid cavity during arthroscopy.

During the intra articular evaluation, the glenoid cavity was viewed via the anterior-superior port and a metallic millimeter ruler designed specifically for this study (Figure 2) was introduced through the posterior port using Burkhart´s method [7]. We used the central area known as the “bare spot”, as a reference point. The tip of the ruler was placed in this spot and the distance between this point to the posterior border and then from the central point to the anterior border (Figure 3) was measured.

### Statistical analysis

Data was collected using Microsoft Office Excel 2003 (Microsoft Corp), and statistical analysis was performed using SPSS 13.0 for Windows (Statistical Package for Social Sciences). The paired Student-*t* test was used to compare: a) the right with the left side, and the dominant with the non-dominant shoulder in Group 1; b) the normal shoulder with the unstable shoulder in Group 2. The Pearson correlation matrix was used to verify the relationship between age and measures of the glenoid cavity in the subjects of Group I. In Group II, this matrix was used to compare the number of dislocations with the measurement of the lower segment. The unpaired Student’s *t* test was used to compare measures between the normal shoulder in the subjects with shoulder instability with the values of the shoulders in the individuals in Group I. We studied the correlation between data found on tomographic studies with the data obtained during arthroscopy. There was agreement between the measurements of segment 6 and the percentage of loss calculated by tomography and during surgery. We applied Kolmogorov-Smirnorv Normalit test for study the rate of 2/6 distribution. The 95% confidence intervals for the mean of the measurements and the intervals with 95% normality were established and all dates were studied on values of p = 0,05.

## Results

Mean values found for Group 1 (normal shoulders) are shown in Table 1 and Table 2 presents the Group 2 data for the unstable shoulders.

**Table 1:** Values found in normal shoulders

	**VS**	**Seg 2**	**Seg 4**	**Seg 6**	**Ratio 2/6**
**Mean**	40.87	17.86	26.50	22.86	0.79
**Median**	41.70	17.20	25.75	22.70	0.80
**MinV**	27.10	13.60	22.40	18.10	0.62
**MaxV**	53.30	24.10	34.10	30.50	0.96
**SD**	5.10	1.98	2.69	2.46	0.09

VS = vertical segment; Seg = segment; MinV = minimum value; MaxV = maximum value; SD = standard deviation.

**Table 2:** Values obtained in shoulders with instability

	**VS**	**Seg 2**	**Seg 4**	**Seg 6**	**Ratio 2/6**
**Mean**	37.33	20.83	24.68	23.07	0.91
**Median**	38.20	21.00	24.40	23.10	0.91
**MinV**	29.60	17.10	19.10	17.80	0.78
**MaxV**	42.30	24.60	31.70	29.90	1.07
**SD**	3.59	1.89	2.66	2.96	0.10

VS = vertical segment; Seg = segment; MinV = minimum value; MaxV = maximum value; SD = standard deviation.

Glenoid cavity measurements found during arthroscopy are listed in Table 3.

**Table 3:** Values measured during arthroscopy (n = 23) Group 2

	**Total IV**	**PC**	**AC**	**Loss**	**Percentage**
**Mean**	24.48	13.61	11.22	2.39	17.57
**Median**	24.00	14.00	11.00	2.00	15.38
**MaxV**	29.00	15.00	14.00	5.00	33.33
**MinV**	18.00	10.00	8.00	0.00	0.00
**SD**	2.47	127	1.68	1.34	9.70

MaxV = maximum value; MinV = minimum value; SD = standard deviation; Total IV = anterior posterior measurement; AC = anterior border to central area; PC = posterior border to central area; Loss = bone loss; Pecentage = percentage of bone loss.

No statistically significant difference was found in the means of the right and left shoulders (p > 0.05) nor between the dominant and non-dominant shoulders (p > 0.05).

Calculations of Pearson´s correlation showed a direct correlation between the following: the Vertical Segment and segment 4, segment 2 and 4, segment 2 and 6, segment 2 and the Ratio 2/6, and segment 4 and 6.

Measurements of segment 4 and segment 6 were inversely related to the Ratio 2/6 (segment 4 P = 0.013, and segment 6 P < 0.001).

Intra class correlations were performed for the right and left sides, and no strong correlation between the sides was found (intra class correlation < 0.75), with the exception of the Vertical Segment, which had a high concordance between the two sides (intra class correlation = 0.97), as shown in Table 4.

**Table 4:** Intra class correlation between right and left sides

**Measurement**	**Intra class correlation (r)**
**Vertical segment**	0.97
**Segment 2**	0.66
**Segment 4**	0.72
**Segment 6**	0.73
**Ratio 2/6**	0.62

The 95% Confidence Intervals (CI) and 95% Normality Intervals (NI) for the Ratio 2/6 were calculated. The 95% CI ranged from 0.77 to 0.88, while the NI ranged from 0.60 to 0.97.

Although the data plotted on the histogram did not appear to follow a normal distribution, the Ratio 2/6 showed a normal distribution when the Kolmogorov-Smirnov test was applied, with p = 0.185.

Comparison of the measurements of segment 6 found on tomography (23.07 ± 2.96) with those taken during arthroscopy, (24.48 ± 2.47) revealed, on average, greater arthroscopy values (p < 0. 001) (Table 5).

**Table 5:** Comparison of measurement of segment 6 on tomography and with arthroscopy (Group 2)

**Measurement**	**Side**	**Mean**	**SD**	**N**	**t-value**	**df**	**p**
Segment 6	Tomography	23.07	2.96	23	−4.63	22	< 0.001
	Arthroscopy	24.48	2.47	23			

SD = standard deviation; N = case number; df = degrees of freedom.

Pearson’s correlation of the two measurements showed a correlation of 0.87 between tomographic and arthroscopic values (r = 0.872 and p < 0.001).

We confirmed that the concordance of the lesion measured by arthroscopy and that calculated using the Ratio 2/6 was 62% with a large variation of 30-82%.

Calculation of the percentage area of the lesion during arthroscopy was done using the “bare spot” as a reference point, as suggested by Burkhart et al. [7]. The percentage of bone loss is calculated using as the numerator, the difference between the anterior portion and the posterior portion, and for the denominator, twice the value of the posterior portion, multiplied by 100%.

$$\begin{array}{l} \mathrm{Percentage}\;\mathrm{of}\;\mathrm{the}\;\mathrm{lesion}=\mathrm{Posterior}\;\mathrm{portion}–\mathrm{Anterior}\;\mathrm{portion}/2\\ \;\times \;\mathrm{posterior}\;\mathrm{portion}\;\times \;100. \end{array}$$

The percentage of erosion based on the 2:6 Ratio found on CT was calculated using the formula:

Percentageofthelesion=100–79/2:6ratio

Concordance of the measurement of segment 6 between tomography and arthroscopy was 75% (Table 6).

**Table 6:** Comparison between segment 6 by tomography and arthroscopy and percentage of lesion measured directly and calculated using the 2/6 ratio (Group 2)

**Measurement**	**Correlation**	**CI (95%)**	
		**Inferior**	**Superior**
Percentage of lesion measured using arthroscopy and segment measurement	0.62	0.30	0.82
Tomography/arthroscopy	0.75	0.50	0.88

CI = Confidence Interval.

## Discussion

We found in this study that the ratio between segment 2 and 6 of the glenoid cavity was 0.79. The 95% Confidence Interval ranged from 0.77 to 0.88, while the Normality Interval ranged from 0.60 to 0.97, which can be used as a normal value when evaluating the degrees of erosion of the anterior border of the glenoid cavity and give us an objective way to conduct the treatment of patients with shoulder instability with erosion of glenoid cavity.

As the basic objective in surgical treatment of shoulder instability is to obtain a stable reduction and prevent future recurrences, the surgical methods should correct not only the anatomical anomalies but also restore the movement arc and muscular force [8].

However, it is impossible to correct. a large impacted fracture of the head of the humerus and extensive erosion of the anterior border of the glenoid cavity, by using arthroscopy because if not treated, are associated with a higher index of recurrence [1].

Several studies have investigated the effects of bone loss from the anterior inferior aspect of the glenoid cavity on shoulder stability [6,9-12].

Our measured values on three dimensional reconstruction using computerized tomography proved similar to values found in the literature [13-16]. This suggests that the 3D CAT exam provides reliable information as to the measurements of the glenoid cavity [17].

Based on anatomic studies, defined parameters are available from control studies, as well as information as to how to measure the normal glenoid cavity. However, with respect to osseous lesions *in vivo*, there are no guidelines as to how to measure the osseous erosions.

At present, there is no standardization or guide on how to measure the size of the osseous lesion. Parameters used to evaluate the erosion of the anterior border of the glenoid cavity vary between different authors and there is no single reliable method described for this measurement [1,6].

Burkhart et al. [7] observed that the formation of an “inverted pear’ is very subjective, and in their study noted that the “bare spot” is in the center of the inferior portion of the cavity, and that the degree of erosion could be obtained based on the difference between the posterior portion and the anterior portion of the “bare spot”.

Ikemoto et al. [18] proposed a CT evaluation method of the amount of osseous erosion using the ratio between the superior and inferior portions of the cavity in which the lesion is localized.

As there is no effective method for measuring erosions, in spite of direct visibility during arthroscopy, there is no consensus as to the viability of the method proposed and advocated by Burkhart et al. [7] which uses the “bare spot” as the center of a circle for the inferior portion of the glenoid cavity [8,19-21].

We showed that the measurement of segment 6 of the inferior portion of the glenoid cavity was greater during arthroscopy than on CT measurements, with values of 24.48 (SD = 2.47) and 23.07 (SD = 2.96) mm, respectively.

Additionally, we found that the measurements had a direct correlation of 0.87 (r = 0.872), which is similar to that found by Griffith et al. [8] of 0.79 (r = 0.79). Direct correlation of these values was 0.75 with a 95% CI between 0.50 and 0.88. Therefore, we can assume that the measurements were done in a standardized manner, as the data correlated positively.

Likewise, the percentage erosion calculated using the 2/6 ratio compared with direct measurement on arthroscopy was low at 0.62.

However, due to the discrepancy between the arthroscopy values and those found during the CT exam, we were unwilling to use the arthroscopy measurements as a “gold standard”. This stemmed from our difficulties in measuring the cavity during arthroscopy, as was pointed out by Griffith et al. [8] and because the method proposed by Burkhart et al. [7] is not exact.

To measure the linear distance in the glenoid cavity, we established a normal parameter in order to have a consistently reliable measurement of lesions of the anterior border of the glenoid cavity. Segment 2 was used as a reference unit, as it is a region in which no change in length occurs during shoulder instability, and compared with the measurements from segment 6 where the erosion occurs.

Although only four measurements were employed (Vertical Segment, segments 2, 4 and 6) in our evaluation, a total of eight measurements were taken to show the homogeneity of the information obtained.

The 2/6 ratio was 0.79, and according to the Kolmogorov-Smirnov test, showed a normal distribution (p = 0.185) with a 95% CI ranging from 0.77 to 0.80. This value was exactly the same as that found by Iannotti et al. [20], who found a value for the ratio of the inferior segment to the superior segment of 1.08 ± 0.01, (29 ± 3.1 mm/23 ± 2.7 mm), which is the same value as 0.79 when one inverts the relationship.

Using this value as a normal value, we calculated that the percentage of bone loss in unstable shoulders could be calculated as: Lesion percentage = 100 – 79/2/6 ratio.

Additionally, we calculated the size of the erosion based on the mean of the value of segment 6 (22.86 mm.); Magnitude of erosion = % erosion × 22.86.

The different ways of calculating bone erosion hamper the comparison of methods. Calculations of the erosion length of 9%, 21%, 34% and 46%, based on the biomechanical studies of Itoi et al. [6], yield values of 2.05; 4.80; 7.76 and 10.51, respectively, which are different from those calculated by Itoi et al. [6], of 2.08; 6.80;10.80 and 14.80. These cuts correspond to the chords of a circle, and have the vertical segment as a reference point, but the values are different when the anterior posterior length of the cavity is used.

These measurements show that segment 2 has a lower value than segment 6 and this confirms the shape of the glenoid cavity as that of a pear [14].

When the erosion compromises the length of segment 6 to the point in which it becomes less than that of segment 2, the configuration of the glenoid cavity changes to that of an ‘inverted pear’ shape. Thus the difference between segment 2 and segment 6 corresponds to the bone loss which leads to an inversion of the configuration of the glenoid cavity. Mean of segment 2 (17.86 mm) – Mean of segment 6 (22.86 mm) = 5 mm, which corresponds to 21.90% of segment 6 and not, as was described by Lo et al. [22], to values of between 25% and 27%.

We showed that the linear variation in measurements of the erosions is very large, from 20-30% of the length of the glenoid cavity. The range in which the authors recommend a reconstruction of the glenoid cavity with a bone graft is from 3.62 mm to 9.15 mm.

However, the percentage that should indicate the use of a bone graft needs to be more precise, as this procedure can have complications [23,24]. On the other hand, lack of an implant may be associated with recurrences of luxation or subluxation, as the stabilizing mechanism of the shoulder has not been corrected.

Due to the complication risk with bone graft, precise indications for their use would be valuable. Pagnani et al. [25] reported that lesions up to 30% of the length of the glenoid cavity treated with capsuloplasty in “open” surgery did not increase the risk of recurrent instability.

Yamamoto et al. [12] showed that erosions of 6 mm, corresponding to 26% of the length of the glenoid cavity, caused considerable articular instability. These values were in agreement with our data (100 × 6 mm/22.86 = 26.20%).

The literature currently suggests the use of a bone graft in the presence of 22-30% erosion. Future clinical studies must provide an objective measurement method for grading the lesion and for the use of an implant, as well as reporting long term follow-up findings on these patients.

A previous diagnosis of erosions of the anterior border of the glenoid cavity is important, as it allows for better surgical planning of the repair to prevent recurrent instability. Arrigoni et al. [26] stressed the importance of arthroscopy done prior to Latarjet, as associated lesions which could compromise the outcomes of treatment can also be diagnosed.

Our study did not result in the determination of a method which could be considered the “Gold Standard” to measure the glenoid cavity, which could have been used as a normal reference during the study. However, the measurements found in our study were similar to those found by other published studies. Thus, we present important findings for the musculoskeletal area [27,28].

## Conclusion

The three-dimensional reconstruction feature of multislice CT imaging software was able to perform measurements of the glenoid cavity, calculating the erosion of the anterior border. The ratio between segment 2 and 6 of the glenoid cavity was 0.79, which can be used as a normal value when evaluating the degrees of erosion of the anterior border of the glenoid cavity. Values found on computerized tomography cannot be used on a comparative basis with measurements obtained during arthroscopy.

## Competing interests

The authors declare that they have no competing interests.

## Authors’ contributions

All authors participated in the acquisition of data and revision of the manuscript. All authors determined the design, interpreted the data and drafted the manuscript. All authors read and gave final approval for the version submitted for publication.
